# CD4^+^T and CD8^+^T Cells in Uterus Exhibit Both Selective Dysfunction and Residency Signatures

**DOI:** 10.1155/2024/5582151

**Published:** 2024-04-22

**Authors:** Shuangpeng Kang, Shuiping Jin, Xueying Mao, BinSheng He, Changyou Wu

**Affiliations:** ^1^Hunan Key Laboratory of the Research and Development of Novel Pharmaceutical Preparations, The Hunan Provincial University Key Laboratory of the Fundamental and Clinical Research on Functional Nucleic Acid, Changsha Medical University, Changsha, China; ^2^Clinical Research Center of Clifford Hospital, Guangzhou, China; ^3^Institute of Immunology, Zhongshan School of Medicine, Sun Yat-sen University, Guangzhou, China

## Abstract

Unlike T cells in other tissues, uterine T cells must balance strong immune defense against pathogens with tolerance to semiallogeneic fetus. Our previous study fully elucidated the characteristics of *γδ*T cells in nonpregnant uterus and the mechanism modulated by estrogen. However, comprehensive knowledge of the immunological properties of *αβ*T (including CD4^+^T cells and CD8^+^T) cells in nonpregnancy uterus has not been acquired. In this study, we fully compared the immunological properties of *αβ*T cells between uterus and blood using mouse and human sample. It showed that most of CD4^+^T cells and CD8^+^T cells in murine uterus and human endometrium were tissue resident memory T cells which highly expressed tissue residence markers CD69 and/or CD103. In addition, both CD4^+^T cells and CD8^+^T cells in uterus highly expressed inhibitory molecular PD-1 and cytokine IFN-*γ*. Uterine CD4^+^T cells highly expressed IL-17 and modulated by transcription factor pSTAT3. Moreover, we compared the similarities and differences between human and murine uterine T cell phenotype. Together, uterine CD4^+^T cells and CD8^+^ cells exhibited a unique mixed signature of T cell dysfunction, activation, and effector function which enabled them to balance strong immune defense against pathogens with tolerance to fetus. Our study fully elucidated the unique immunologic properties of uterine CD4^+^T and CD8^+^T cells and provided a base for further investigation of functions.

## 1. Introduction

According to the constitution of T cell receptor (TCR), T cells can be divided into *αβ*T cells and *γδ*T cells. *αβ*T cells are the major T cell subsets of immune system and most mature *αβ*T cells were CD4^+^ or CD8^+^ single positive cells [1, 2]. When encounter the pathogen, antigen-specific T cells are activated, proliferate, and expand extensively. Most effector T cells undergo apoptosis in the contraction phase of the response. However, a minor fraction will eventually differentiate into memory T cells which could be parsed into effector memory T cells (T_EM_), central memory T cells (T_CM_) or tissue resident memory T (T_RM_) cells according to their homing characteristics and effector functions. Different from circulating T cells which survey the blood and lymph for pathogens, T_RM_ cells do not recirculate and persist in peripheral tissues in the long term. T_RM_ population have been detected in many tissues such as reproductive tract, skin, and small intestine and play critical roles in local immunity [3–6].

Compared with other mucosal surfaces, uterine mucosal immune system is quite unique because it must balance strong immune defense against pathogens with tolerance to semiallogeneic fetus. The nonpregnant endometrium contains a well-marked number of lymphocytes, mainly T cells (6%–60%) and NK cells (25%–85%), which constantly change during the menstrual cycle. Immune cells in endometrium appear as lymphoid aggregates surrounded by a core of B cells and lined by CD8^+^T cells and macrophages. Dysfunctions of endometrial or decidual immune cells are reported to be closely related with infertility, miscarriage, and other pregnant complications [7–9].

Our previous study [10, 11] fully elucidated the phenotypic and functional properties of uterine *γδ*T cells and found that *γδ*T cells were greatly enriched in human and murine uterus which abundantly expressed IL-17 and were the main source of IL-17 in uterus. Furthermore, estrogen could further stimulate the production of IL-17 through promoting the expression of transcription factor IRF-4 and migration to uterus of *γδ*T cells. Following previous studies, here, we fully elucidated the immunology characteristics of uterine CD4^+^T and CD8^+^T cells. Our study provided a base for further investigation on the role of uterine T cells in the development of autoimmune diseases in women.

## 2. Materials and Methods

### 2.1. Subjects and Samples

Peripheral blood and endometrium tissues were collected from 20 patients (age 32–49 years old) with uterine prolapse who underwent hysterectomy at Third Affiliated Hospital of Sun Yat-sen University. Patients who had been previously diagnosed with HIV, hepatitis B, or hepatitis C, cancer, endometritis, endometrial polyps, uterine fibroids or who had a history of autoimmune diseases or immune deficiency were ruled out of the study. The experimental protocol was approved by the ethics committee of Changsha Medical University. Informed consents were obtained from all patients before sample collection.

### 2.2. Animals

Aged 6–8 weeks female C57BL/6 mice were purchased from the Laboratory Animal Center of Sun Yat-sen University and reared under specific pathogen-free conditions. Mice were matched for age and weight in each experiment. All animal studies approved and supervised by the ethics committee of Changsha Medical University.

### 2.3. Cell Isolation

As previously described [10], cardiac perfusion was used to remove blood in uterus. Murine uterus and human endometrium were cut into 1–2 mm^3^ pieces and then transferred to 5–10 mL digestion medium which contained 2 mg/mL collagenase I (Sigma–Aldrich, USA) and 10 mg/mL DNase I (Sigma–Aldrich, USA) in RPMI-1640 for 1 hr at 37°C in a shaker. The digested samples were filtered through a 100-*μ*m cell strainer (BD Bioscience, USA). Percoll (GE Healthcare, USA) density gradient centrifugation were used to further isolate lymphocytes in uterus and washed twice with complete RPMI-1640 medium. Finally, cells were counted and suspended at 1 × 10^6^ cells/mL in complete RPMI-1640 medium.

Blood was obtained through eye socket bleeding. Diluted blood was loaded onto Ficoll–Hypaque (Tianjin HaoYang Biological Manufacture, China) and isolated by density gradient centrifugation according to manufacture protocol. The cells were counted and suspended at 1 × 10^6^ cells/mL in complete RPMI-1640 medium.

### 2.4. Cell Culture

For cytokines and transcription factors staining, isolated cells above were stimulated for 6 hr with 20 ng/mL PMA (Sigma–Aldrich) plus 1 *μ*g/mL Ionomycin (Sigma–Aldrich) in the existence of 10 *μ*g/mL brefeldin A (Sigma–Aldrich) at 37°C with 5% CO_2_.

### 2.5. mAbs

The following antimouse mAbs were purchased from BD Bioscience: PE-CF594-labeled anti-CD3 (clone: 145-2C11), anti-PD-1(clone: J43); FITC-labeled anti-CD3 (clone: 145-2C11), anti-*γδ*T (clone: GL3), anti-CD107a (clone: 1D4B), anti-CD4 (clone: H129.19); APC labeled anti-CD8 (clone: 53-6.7), anti-IFN-*γ* (clone: XMG1.2), anti-CD4 (clone: RM4-5), anti-CD25 (clone: PC61), anti-TNF-*α* (clone: MP6-XT22), anti-LAG-3 (clone: C9B7W); PE-labeled anti-IL-17 (clone: TC11-18H10), anti-CD103 (clone: M290), anti-TIM-3 (clone: RMT3-23), anti-CTLA-4 (clone:UC10-4F10-11), anti-CXCR3 (clone: CXCR3-173), anti-CCR5 (clone:C34-3448), anti-ROR*γ*t (clone: Q31-378), anti-pSTAT3 (clone: 4/P-STAT3); Percp-cy5.5-labeled anti-CD8 (clone: 53-6.7), anti-CD27 (clone: LG.3A10), anti- CD44 (clone: IM7); PE-cy7 labeled anti-CD69 (clone: H1.2F3), anti-IFN-*γ* (clone: XMG1.2); and Apc-cy7-labeled anti-IL-17 (clone: TC11-18H10). The following antimouse mAbs were purchased from biolegend: PE-labeled anti-GranzymeB (clone: QA16A02), anti-CX3CR1 (clone: QA16A03); PE-cy7-labeled anti-CD103 (clone:2E7), anti-CCR2 (clone: SA203G11), anti-CCR4 (clone: 2G12), and anti-CCR6 (clone: 29-2L17).

The following antihuman mAbs were purchased from BD Bioscience: PE-CF594-labeled anti-CD3 (clone: UCHT1); FITC-labeled anti-CD4 (clone: M-T47), anti-IFN-*γ* (clone: B27); PE-labeled anti-CD103 (clone: Ber-ACT8), anti-IL-17 (clone: SCPL1362); Percp-cy5.5-labeled anti-CD8 (clone: SK1); Alexa Fluor 700-labeled anti-CD45RO (clone: UCHL1); and PE-cy7-labeled anti-CD69 (clone: FN50).

### 2.6. Flow Cytometry

As previously described [12], cells were washed with PBS buffer containing 0.1% BSA and 0.05% sodium azide, and finally suspended in 100 *μ*L staining buffer. For phenotyping, cells were stained with respective mAbs in the dark for 30 min at 4°C. For staining of intracellular cytokines, cells after staining for the phenotypes described above were fixed with 4% paraformaldehyde for 8 min and then permeabilized with PBS buffer which contained 0.1% saponin, 0.1% BSA and 0.05% NaN_3_ overnight at 4°C or room temperature for 2 hr. Cells were washed twice and stained with corresponding mAbs in dark for 30 min. For the staining of transcription factors, cells after staining for the phenotypes described above were fixed and permeabilized with permeabilization/fixation buffer (BD Bioscience, USA) according to manufacture protocol, and then stained with indicated cytokines and transcription factors mAbs. Cells were assayed by FACS Aria II (BD Bioscience, USA). The flow cytometry data were analyzed with FlowJo10 (Tree Star, USA).

### 2.7. Statistical Analysis

All statistical analysis was performed with GraphPad Prism6 (USA). Unpaired Student's *t*-test was used to compare two groups. Data were presented as the mean ± standard error of mean (SEM). NS, no significance;  ^*∗*^*P* < 0.05;  ^*∗∗*^*P* < 0.01;  ^*∗∗∗*^*P* < 0.001;  ^*∗∗∗∗*^*P* < 0.0001.

## 3. Results

### 3.1. There Were Striking Differences in the Subsets of T Cells in Uterus and Blood

**Figure 1:**
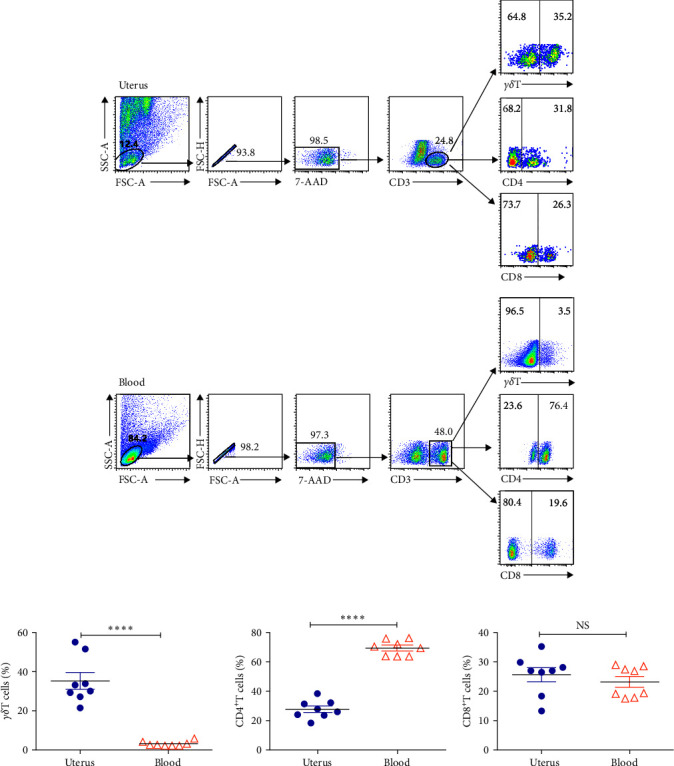
The proportion of different subsets of T cells in uterus and blood. Cells isolated from uterus and blood were stained with anti-CD3, anti-CD4, anti-CD8, and anti-*γδ*T mAb and detected the percentages of three subsets of T cells using flow cytometry. Live and single-celled CD3^+^T cells were gated and subsequently analyzed the percentages of CD4^+^T, CD8^+^T, and *γδ*T cells from uterus and blood. (a) Statistical graph for the percentages of three T cells subsets were shown. (b) Data were presented as mean ± SEM and compared with the Student's *t*-test. NS, no significance;  ^*∗∗∗∗*^*P* < 0.0001.

Cells isolated from uterus and blood were stained with anti-CD3, anti-CD4, anti-CD8, and detected by flow cytometry to investigate the percentages of T cell subsets. The gating strategy showed that live and single-celled CD3^+^T cells were gated (Figure 1(a)). The results showed that the proportion of CD3^+^T cells was significantly lower in uterus than that in blood. Further analysis of the percentages of three subsets of T cells, namely *γδ*T cells, CD4^+^ T cells, and CD8^+^ T cells, found that the percentages of *γδ*T cells (35.2%) was markedly higher than that in blood (3.03%, *P* < 0.0001). By contrast, the proportion of CD4^+^T cells in uterus (27.4%) was much lower than in blood (69.2%, *P* < 0.0001). No difference was observed in the proportion of CD8^+^T cells from uterus and blood (Figures 1(a) and 1(b)).

### 3.2. Most of CD4^+^T Cells and CD8^+^T Cells in Uterus Were Tissue Resident Memory T Cells

**Figure 2:**
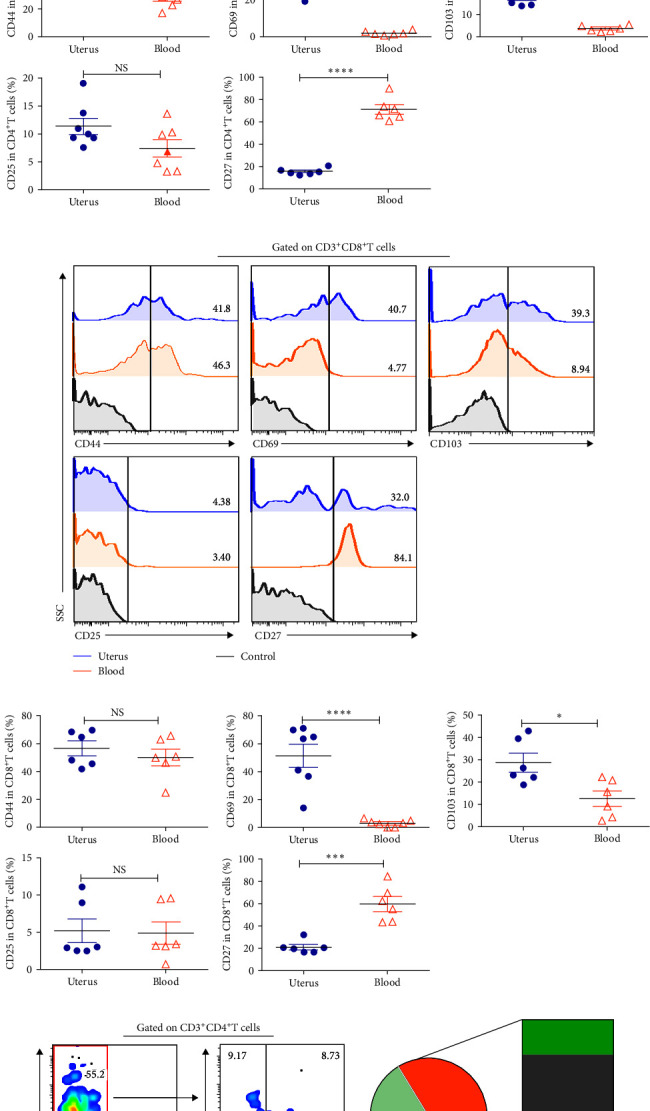
Expression of memory and residence markers on CD4^+^T cells and CD8^+^T cells from uterus and blood. Cells from uterus and blood were isolated and stained with anti-CD44, CD69, CD103, CD25, and CD27 mAbs. Gated on CD3^+^CD4^+^T (a, b) and CD3^+^CD8^+^T cells (c, d), and then compare the expression of CD44, CD69, CD103, CD25, and CD27 in uterus and blood. Representative dot plots and pie chart (*n* = 6) showed that gated on uterine CD3^+^CD4^+^CD44^+^ memory T cells (e) and CD3^+^CD8^+^T CD44^+^ memory T cells (f), and then detected the proportion of non-T_RM_ (CD69^−^CD103^−^) and T_RM_ (CD69^+^CD103^+^, CD69^+^CD103^−^, and CD69^−^CD103^+^) cells. Data were shown as mean ± SEM and compared with the Student's *t*-test. NS, no significance;  ^*∗*^*P* < 0.05;  ^*∗∗*^*P* < 0.01;  ^*∗∗∗*^*P* < 0.001;  ^*∗∗∗∗*^*P* < 0.0001.

T cells may have a unique phenotype, chemokine receptors, and cytokine expressions, representing the diversity of its function. To identify the phenotype of CD4^+^T cells and CD8^+^T cells in uterus, we analyzed the expression of molecules associated with memory differentiation (CD44), tissue residency (CD69 and CD103), activation (CD25), and costimulation (CD27) by FACS. The results showed that about half of the CD4^+^T cells and CD8^+^T cells were memory T cells which highly expressed CD44. Uterine CD4^+^T cells and CD8^+^T cells expressed obviously higher levels of CD69 and CD103 than that of blood. By contrast, the expression of CD27 on CD4^+^T cells and CD8^+^T cells from uterus was clearly lower than that from blood, and the levels of CD25 on CD4^+^T cells and CD8^+^T cells had no statistical significance between uterus and blood (Figure 2(a)–2(d)). Numerous experiments have confirmed and characterized T_RM_ phenotype cells expressing CD69 and/or CD103 using both mouse and human samples. CD69 is expressed by most of CD4^+^ and CD8^+^ T_RM_ cells, whereas CD103 is only expressed by certain subsets of CD8^+^ T_RM_ cells [5]. Here, we used putative T_RM_ markers CD69 and CD103 to define them. Gated on uterine CD44^+^ memory T cells, 61.3% CD4^+^T and 77.4% CD8^+^T cells in uterus were T_RM_ cells and could be classified into three groups according to the expression of CD69 and CD103: CD69^+^CD103^+^, CD69^+^CD103^−^, or CD69^−^CD103^+^. Taken together, these data suggested that a significant portion of CD4^+^T cells and CD8^+^T cells in uterus were T_RM_ cells.

### 3.3. CD4^+^T Cells and CD8^+^T Cells in Uterus Had a Distinct Profile of Chemokine Receptors Expressions

**Figure 3:**
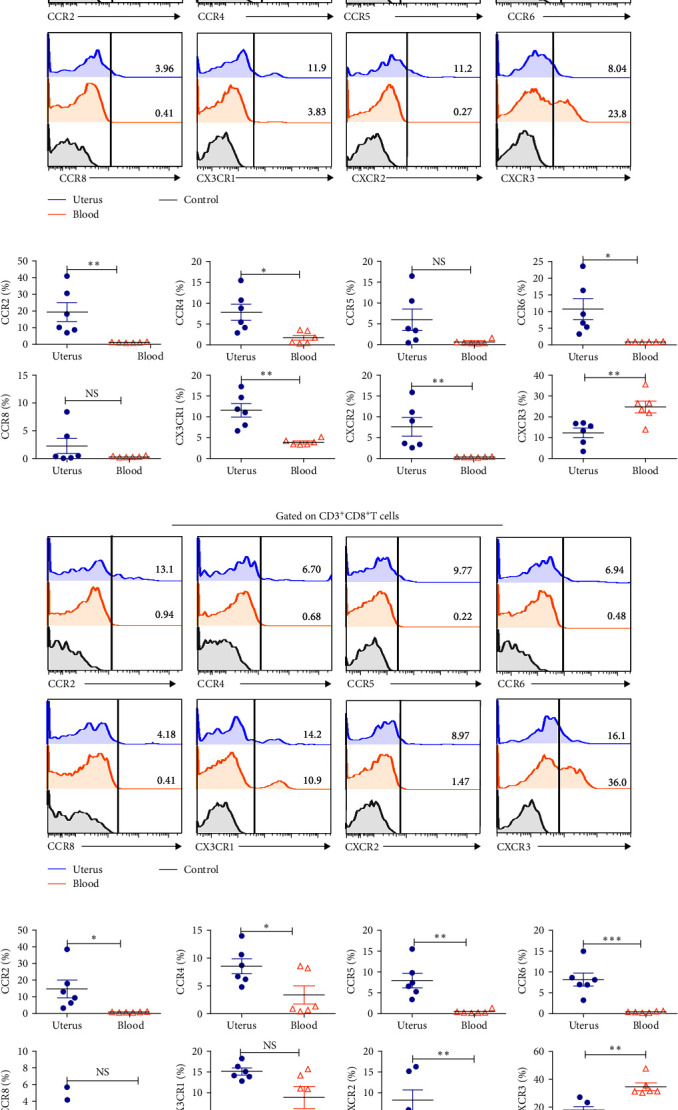
The expression of chemokine receptors on CD4^+^T cells and CD8^+^T cells from uterus and blood. Gated on CD3^+^CD4^+^T (a, b) and CD3^+^CD8^+^T cells (c, d), and then compare the expression of chemokine receptors including CCR2, CCR4, CCR5, CCR6, CCR8, CX3CR1, CXCR2, and CXCR3 in uterus and blood. Data were presented as mean ± SEM and compared with the Student's *t*-test. NS, no significance;  ^*∗*^*P* < 0.05;  ^*∗∗*^*P* < 0.01;  ^*∗∗∗*^*P* < 0.001.

T cells in different tissues have a distinct profile of chemokine receptors expressions, indicating different migration patterns of lymphocytes to lymphoid and nonlymphoid tissues. We further investigated the profile of chemokine receptors expression in uterine CD4^+^T cells and CD8^+^T. It found that uterine CD4^+^T cells expressed slightly higher levels of CCR2, CCR4, CCR6, CX3CR1, and CXCR2, whereas expressed lower levels of CXCR3 than of blood. The expression of CCR5 and CCR8 was not statistically different (Figures 3(a) and 3(b)). Further analysis found that the expression of CCR2, CCR4, CCR5, CCR6, and CXCR2 on uterine CD8^+^T cells was a little higher than that of blood, whereas the expression level of CXCR3 was lower than of blood. The levels of CCR8 and CX3CR1 on CD8^+^T cells had no statistical significance between uterus and blood (Figures 3(c) and 3(d)).

### 3.4. Uterine CD4^+^T Cells and CD8^+^T Cells Expressed Markedly Higher Levels of PD-1 than in Blood, but Not Other Inhibitory Receptors

**Figure 4:**
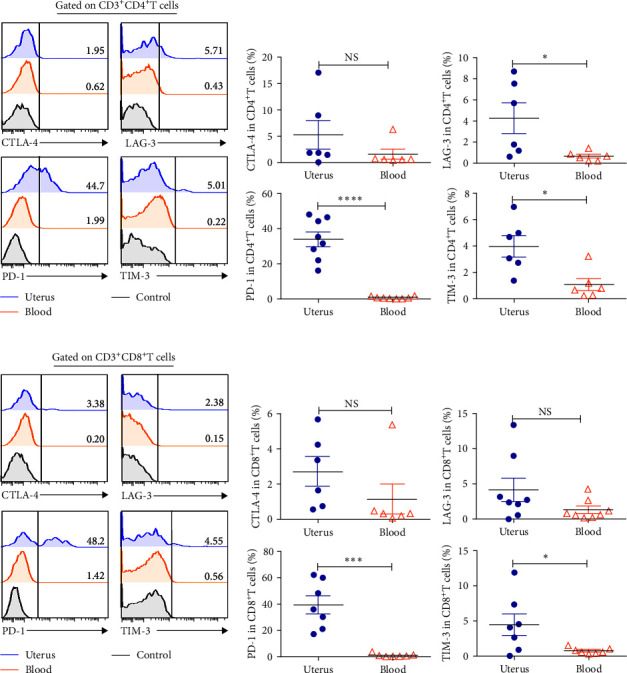
Expression of inhibitory receptor on CD4^+^T cells and CD8^+^T cells from uterus and blood. Representative graph and statistical charts showed that gated on CD3^+^CD4^+^T (a, b) and CD3^+^CD8^+^T cells (c, d), and then compare the expression of inhibitory receptor including CTLA-4, LAG-3, PD-1, and TIM-3 in uterus and blood. Data were presented as mean ± SEM and compared with the Student's *t*-test. NS, no significance;  ^*∗*^*P* < 0.05;  ^*∗∗∗*^*P* < 0.001;  ^*∗∗∗∗*^*P* < 0.0001.

T cell dysfunction has been confirmed in various cancers and chronic infections. Dysfunctional T cells lose the ability to robustly response to pathogens, and often express multiple and high levels of inhibitory receptors (such as, PD-1, LAG-3, CTLA-4, and TIM-3) [13]. Here, we found that 34.3% uterine CD4^+^T cells expressed PD-1, markedly higher than that in blood (0.83%, *P* < 0.0001) (Figures 4(a), and 4(b)). Meanwhile, uterine CD8^+^T cells (39.2%) expressed greatly higher PD-1 than that of blood (1.00%, *P* < 0.001) (Figures 4(c) and 4(d)). Both uterine CD4^+^T cells and CD8^+^T cells and blood expressed minimal levels of CTLA-4, LAG-3, and TIM-3 (Figure 4(a)–4(d)).

### 3.5. Uterine CD4^+^T and CD8^+^T Cells Had the Capacity to Produce Effector Molecules

**Figure 5:**
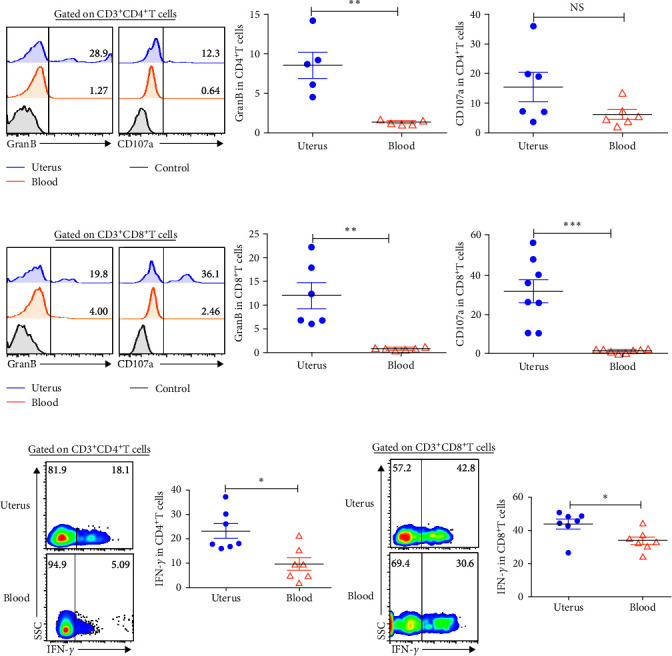
Uterine CD4^+^T and CD8^+^T cells highly expressed cytotoxic molecules and IFN-*γ*. Isolated cells from uterus were stained with phenotype markers, anti-GranzymeB, anti-CD107a, and Anti-IFN-*γ* mAbs. Representative and statistical charts showed that gated on CD3^+^CD4^+^T (a) and CD3^+^CD8^+^T cells (b), and then compared to the expression of cytotoxic molecules including GranzymeB and CD107a in uterus and blood. Isolated uterine cells were stimulated with or without PMA and Ionomycin in the existence of BFA for 6 hr to detect the expression of IFN-*γ*. The expression of IFN-*γ* on CD3^+^CD4^+^T (c) and CD3^+^CD8^+^T cells (d) were shown. Data were presented as mean ± SEM and compared with the Student's *t*-test. NS, no significance;  ^*∗*^*P* < 0.05;  ^*∗∗*^*P* < 0.01;  ^*∗∗∗*^*P* < 0.001.

The expression of cytotoxic molecules Granzyme B and CD107a were assessed by FACS to determine the killing ability of CD4^+^T and CD8^+^T cells from uterus and blood. The results indicated that uterine CD4^+^T cells (13.8%) expressed higher levels of Granzyme B than blood (1.45%, *P* < 0.01), and there was no statistically significant difference in CD107a expression (Figure 5(a). Moreover, uterine CD8^+^T cells expressed higher levels of Granzyme B and CD107a than in blood (Figure 5(b)). To further confirm the ability to produce cytokines, isolated cells were stimulated with or without PMA and Ionomycin in the existence of BFA for 6 hr and then checked for the expression of IFN-*γ* by flow cytometry. The results demonstrated that compared with blood, uterine CD4^+^T cells and CD8^+^T cells expressed higher levels of IFN-*γ* (Figures 5(c) and 5(d)). Overall, compared with blood, CD4^+^T cells and CD8^+^T cells in uterus had greater cytotoxic activity and IFN-*γ* expression.

### 3.6. Uterine CD4^+^T Cells Highly Expressed IL-17 and Modulated by Transcription Factor pSTAT3

**Figure 6:**
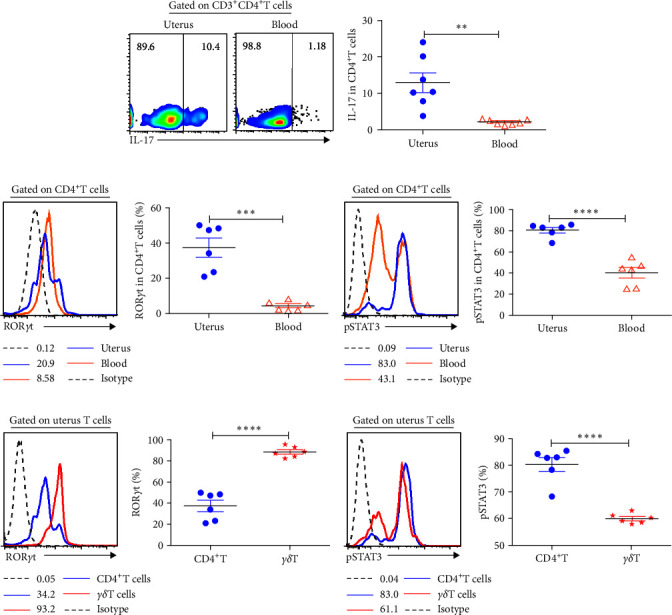
Uterine CD4^+^T cells expressed IL-17 and pSTAT3 abundantly. Isolated uterine cells were stimulated with or without PMA and Ionomycin in the existence of BFA for 6 hr and then stained with phenotype markers, anti-IL-17, anti-ROR*γ*t, and anti-pSTAT3. The expression of IL-17 (a), ROR*γ*t (b), and pSTAT3 (c) on CD3^+^CD4^+^T from uterus and blood were shown. The comparison of the expression of ROR*γ*t (d) and pSTAT3 (e) between uterine CD4^+^T and *γδ*T^+^ cells was shown. Data were presented as mean ± SEM and compared with the Student's *t*-test.  ^*∗∗*^*P* < 0.01;  ^*∗∗∗*^*P* < 0.001;  ^*∗∗∗∗*^*P* < 0.0001.

To evaluate the expression of IL-17, cells isolated from uterus and blood were stimulated with or without PMA and Ionomycin for 6 hr in the existence of BFA. It showed that the frequencies of uterine CD4^+^T cells expressing IL-17 were significantly higher (12.9%) than that of blood (2.10%, *P* < 0.01) (Figure 6(a)). To explore the mechanism modulating the expression of IL-17 by uterine CD4^+^T cells, we evaluated the expression of ROR*γ*t and pSTAT3 which were key transcription factor modulating IL-17 production. The data showed that uterine CD4^+^T cells expressed higher levels of transcription factor ROR*γ*t and pSTAT3, especially pSTAT3, than in blood (Figures 6(b) and 6(c)). Different from uterine *γδ*T cells which stable expressed ROR*γ*t (88.6%), CD4^+^T cells in uterus showed much less expression of it (37.4%, *P* < 0.0001) (Figure 6(d)). Conversely, uterine CD4^+^T cells expressed pSTAT3 abundantly (Figure 6(e)).

### 3.7. The Similarities and Differences between Human and Murine Uterine T Cell Phenotype

**Figure 7:**
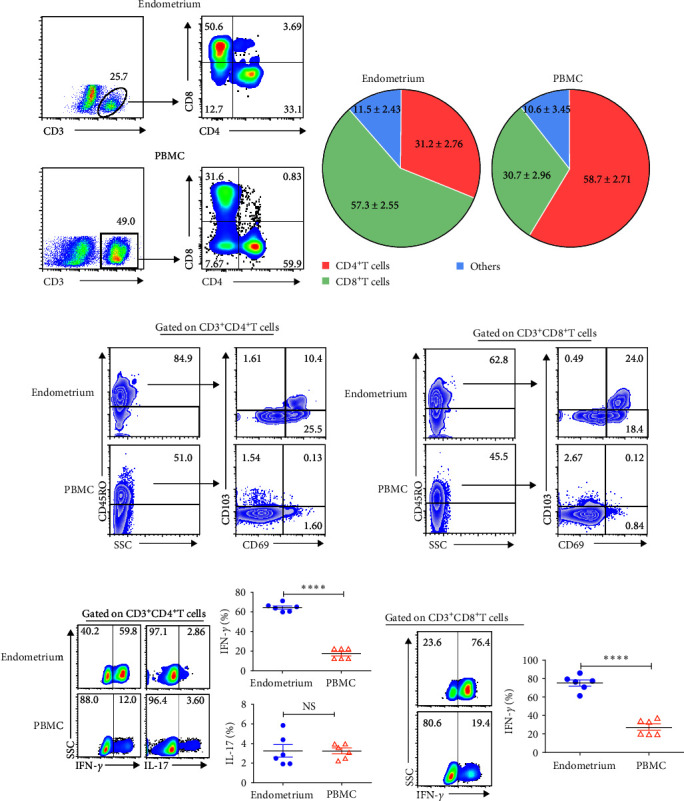
The immunological characteristics of CD4^+^T and CD8^+^T cells from human endometrium and blood. Isolated cells were incubated with respective mAbs and the percentages of T cell subsets were detected by flow cytometry. The representative dot plots (a) and pie chart (*n* = 6) (b) for the frequencies of CD4^+^T, CD8^+^T, and other T cells in human endometrium and PBMC were shown. The representative dot plots showed the frequencies of CD45RO, CD69, and CD103-expressing CD4^+^T cells (c) and CD8^+^T cells (d) from human endometrium and blood. Data were representative of three independent experiments. Isolated cells from human endometrium were stimulated with or without PMA and Ionomycin in the existence of BFA for 6 hr and then assessed the expression of IFN-*γ* and IL-17. The frequencies of IFN-*γ* and IL-17-expressing CD4^+^T cells (e) and CD8^+^T cells (f) from human endometrium and blood were shown. Data were presented as mean ± SEM and compared with the Student's *t*-test. NS, no significance;  ^*∗∗∗∗*^*P* < 0.0001.

Numerous study points to T_RM_ cells play a critical role in local tissue immunity. However, much of this knowledge comes from murine researches. Little is known about T_RM_ cells in human endometrium due to a variety of biological variables such as hormonal changes, sexual activity, and age which can influence their abundance and function [14]. To further determine similarities and discrepancies in immunological characteristics of human and mouse CD4^+^T and CD8^+^T cells, we recruited 20 individuals in the luteal phase and collected endometrium samples. It showed that 57.3% of T cells were CD8^+^T cells which were the major subset in human endometrium. In contrast, the frequencies of CD4^+^T cells in endometrium (31.2%) was clearly lower than in blood (58.7%) (Figures 7(a) and 7(b)). Consistent with results in murine uterus, CD4^+^T cells and CD8^+^T cells highly expressed memory marker CD45RO and residence markers CD69 and CD103 (Figures 7(c) and 7(d)). To assess the expression of IL-17 and IFN-*γ*, cells isolated from human endometrium and blood were stimulated with or without PMA and Ionomycin for 6 hr in the existence of BFA. It showed that the expression of IL-17 on CD4^+^T cells from endometrium and blood had no significant difference (Figure 7(e)). By contrast, compared with blood, both CD4^+^T cells and CD8^+^T cells from endometrium highly expressed IFN-*γ* (Figures 7(e) and 7(f)). In short, there were some differences in immunological characteristics of CD4^+^T cells and CD8^+^T cells between human and mouse uterus.

## 4. Discussion

To establish a successful pregnancy, immune cells at the maternal–fetal interface must combine the competitive requirements for immune tolerance to semiallogeneic fetus and immune defense against pathogens. As yet, the function of decidual T cells in pregnancy has become evident [15–18]. However, few literature on T cells of nonpregnant uterine has been published. In this study, we found that uterine CD4^+^T and CD8^+^T cells exhibited a mixed signature of T cell dysfunction, activation, and effector function. Uterine CD4^+^T and CD8^+^T cells highly expressed residence markers CD69 and CD103, inhibitory molecular PD-1 and cytokine IFN-*γ*. Uterine CD4^+^T cells highly expressed IL-17 and modulated by transcription factor pSTAT3. Moreover, we found that there were some differences in immunological characteristics of CD4^+^T and CD8^+^T cells between human and murine uterus.

Different from peripheral blood, the frequencies of *γδ*T cells, CD4^+^T cells, and CD8^+^T cells were roughly equal in murine uterus, about 30% each. However, in human endometrium, CD8^+^T cells were the richest T cell subsets, accounting for 57.3%. In human endometrium, a mass of CD8^+^T cells surrounded a core B cells to form lymphoid aggregates [9]. Despite the classic cytotoxic nature of CD8^+^T cells in infection immunity, uterine CD8^+^T cells are key mechanism of fetus immune tolerance, which alter in pathological process [19–21]. T_RM_ is a newly discovered effector memory cell which act as sentinels in peripheral tissues. Once established, T_RM_ cells provide rapid and robust immune responses to pathogens entering through local tissues due to their unique properties, such as the ability to colonize barrier tissues, survive for a long time, and deploy ready effector functions. CD69 and CD103 are two key markers expressed by most of T_RM_ cells which contribute to their residency [5, 22]. Our study indicated that both CD4^+^T cells and CD8^+^T cells in murine uterus and human endometrium expressed markedly high levels of CD69 and CD103 which was indicative of the resident character of these cells. We used putative T_RM_ markers CD69 and CD103 to define them and found that 61.3% CD4^+^T cells and 77.4% CD8^+^T cells were T_RM_ cells in murine uterus. The periodic remodeling of the functional layer of the endometrium during menstrual cycle seems to conflict with the definition of tissue retention. However, some investigations have revealed the stable persistence of T_RM_ as well as ILC1 and NK cells in endometrium during interpregnancy intervals [3, 23–25].

Intriguingly, most uterine *γδ*T cells expressed activation marker CD25, about 70% [10] but not CD4^+^T and CD8^+^T cells. CD4^+^T and CD8^+^T cells in uterus expressed much lower levels of costimulatory receptors CD27 which is involved in the regulation of T cell activation, especially in T cell memory [26, 27]. These may indicate uterine *γδ*T cells and *αβ*T cells play different roles in intrauterine immune microenvironment with the latter favoring maternal–fetal tolerance. T cell dysfunction has been confirmed in various cancers and chronic infections which is usually related to the low control efficiency of continuous antigens. Dysfunctional T cells lose the ability to robustly response to pathogens, and often express multiple and high levels of inhibitory receptors, and cannot produce sufficient effector cytokines or cytotoxic molecules [13, 28, 29]. Here, the increased expression of PD-1 together with low expression of activation markers may suggest that uterine CD4^+^T and CD8^+^T cells have reduced the effector capacity to maintain immune tolerance to fetal. Meanwhile, the ability of them to upregulate Granzyme B and CD107a expression and produce proinflammatory cytokines (IFN-*γ*, IL-17) upon activation suggested these cells were not permanently inhibited and retained the ability to confront incoming pathogens. Together, these results indicated that the selective dysfunction of uterine CD4^+^T and CD8^+^T cells could balance immune defense and maternal–fetal immune tolerance.

IL-17 has attracted wide attention for its protective effect to eliminate extracellular bacteria and fungi and pathogenic role in autoimmune disease [30, 31]. However, different studies showed conflicting results as to whether IL-17 is good or bad for successful pregnancy. An elevated Treg/Th17 ratio is considered to be a key cause of successful pregnancy, which is blunted in recurrent pregnancy loss and pre-eclampsia [18]. Some studies have reported that IL-17 could enhance the proliferation and invasion of trophoblast cells, and prevent apoptosis of trophoblast cells during the first trimester of pregnancy [10, 32]. In a previous study, we found that *γδ*T cells were the major source of IL-17 in uterus and produced IL-17 three times more frequently than CD4^+^T cells [10]. ROR*γ*t and STAT3 were the most critical transcription factors regulating the formation of IL-17 [33–36]. Uterine CD4^+^T cells abundantly expressed pSTAT3, but expressed low levels of ROR*γ*t. Conversely, nearly 90% of uterine *γδ*T cells expressed ROR*γ*t but a lower proportion expressed pSTAT3. The production of IL-17 of uterine CD4^+^T cells and *γδ*T cells might be modulated by pSTAT3 and ROR*γ*t, respectively. Finally, it has to be noted that there were some differences in proportion and cytokine production of CD4^+^T cell and CD8^+^T cells between human and murine uterus.

Numerous investigations focused on how decidual T cells are involved in maternal–fetal tolerance. T cells in nonpregnancy uterus have not received much attention in the past. Here, we systematically evaluated the immunological properties of uterine CD4^+^T and CD8^+^T cells, which exhibited a unique mixed signature of T cell dysfunction, activation, and effector function that enable them to balance strong immune defense against pathogens with tolerance to a fetus. This study together with our previous study in uterine *γδ*T cells provided important information related to function of uterine T cells which may influence various areas of clinical immunology, especially in pregnancy and autoimmune disease.

## Data Availability

The datasets supporting the present study can be available from the corresponding author upon request.

## References

[1] Brenner M. B., McLean J., Dialynas D. P. (1986). Identification of a putative second T-cell receptor. *Nature*.

[2] Ribot J. C., Lopes N., Silva-Santos B. (2021). *γδ*T cells in tissue physiology and surveillance. *Nature Reviews Immunology*.

[3] Yüzen D., Arck P. C., Thiele K. (2022). Tissue-resident immunity in the female and male reproductive tract. *Seminars in Immunopathology*.

[4] Okła K., Farber D. L., Zou W. P. (2021). Tissue-resident memory T cells in tumor immunity and immunotherapy. *Journal of Experimental Medicine*.

[5] Parga-Vidal L., van Aalderen M. C., Stark R., van Gisbergen K. P. J. M. (2022). Tissue-resident memory T cells in the urogenital tract. *Nature Reviews Nephrology*.

[6] Mao X., Chen Y., Lu X. (2023). Tissue resident memory T cells are enriched and dysfunctional in effusion of patients with malignant tumor. *Journal of Cancer*.

[7] Lee S. K., Kim C. J., Kim D.-J., Kang J.-H. (2015). Immune cells in the female reproductive tract. *Immune Network*.

[8] Agostinis C., Mangogna A., Bossi F., Ricci G., Kishore U., Bulla R. (2019). Uterine immunity and microbiota: a shifting paradigm. *Frontiers in Immunology*.

[9] Yeaman G. R., Collins J. E., Fanger M. W., Wira C. R. (2001). CD8^+^ T cells in human uterine endometrial lymphoid aggregates: evidence for accumulation of cells by trafficking. *Immunology*.

[10] Kang S., Wu Q., Huang J. (2021). Tissue resident memory *γδ*T cells in murine uterus expressed high levels of IL-17promoting the invasion of trophocytes. *Frontiers in Immunology*.

[11] Kang S. P., Wu Q. L., Wan S. Q., Wu C. Y. (2022). Estrogen promoted the production of IL-17 by tissue resident memory *γδ*T cells in murine uterus via transcription factor IRF-4. *Faseb Journal*.

[12] Cao J., Chen C., Wang Y., Chen X., Chen Z., Luo X. (2016). Influence of autologous dendritic cells on cytokine-induced killer cell proliferation, cell phenotype and antitumor activity in vitro. *Oncology Letters*.

[13] Philip M., Schietinger A. (2022). CD8^+^ T cell differentiation and dysfunction in cancer. *Nature Reviews Immunology*.

[14] Lund J. M., Hladik F., Prlic M. (2023). Advances and challenges in studying the tissue- resident T cell compartment in the human female reproductive tract. *Immunological Reviews*.

[15] Wang S. C., Li Y. H., Piao H. L. (2015). PD-1 and Tim-3 pathways are associated with regulatory CD8^+^ T-cell function in decidua and maintenance of normal pregnancy. *Cell Death & Disease*.

[16] Liu L., Huang X., Xu C. (2020). Decidual CD8^+^T cells exhibit both residency and tolerance signatures modulated by decidual stromal cells. *Journal of Translational Medicine*.

[17] van der Zwan A., Bi K., Norwitz E. R. (2018). Mixed signature of activation and dysfunction allows human decidual CD8^+^ T cells to provide both tolerance and immunity. *Proceedings of the National Academy of Sciences of the United States of America*.

[18] Liu S., Diao L., Huang C., Li Y., Zeng Y., Kwak-Kim J. Y. H. (2017). The role of decidual immune cells on human pregnancy. *Journal of Reproductive Immunology*.

[19] Hardardottir L., Bazzano M. V., Glau L. (2021). The new old CD8^+^ T cells in the immune paradox of pregnancy. *Frontiers in Immunology*.

[20] Lager S., Sovio U., Eddershaw E. (2020). Abnormal placental CD8^+^T-cell infiltration is a feature of fetal growth restriction and pre-eclampsia. *The Journal of Physiology*.

[21] Wang S. C., Sun F. R., Li M. D. (2019). The appropriate frequency and function of decidual Tim-3^+^CTLA-4^+^CD8^+^ T cells are important in maintaining normal pregnancy. *Cell Death & Disease*.

[22] Yang K., Kallies A. (2021). Tissue-specific differentiation of CD8^+^ resident memory T cells. *Trends in Immunology*.

[23] Southcombe J. H., Mounce G., McGee K. (2017). An altered endometrial CD8 tissue resident memory T cell population in recurrent miscarriage. *Scientific Reports*.

[24] Filipovic I., Chiossone L., Vacca P. (2018). Molecular defnition of group 1 innate lymphoid cells in the mouse uterus. *Nature Communications*.

[25] Gamliel M., Goldman-Wohl D., Isaacson B. (2018). Trained memory of human uterine NK cells enhances their function in subsequent pregnancies. *Immunity*.

[26] Hendriks J., Gravestein L. A., Tesselaar K., van Lier R. A. W., Schumacher T. N. M., Borst J. (2000). CD27 is required for generation and long-term maintenance of T cell immunity. *Nature Immunology*.

[27] Honikel M. M., Olejniczak S. H. (2022). Co-stimulatory receptor signaling in CAR-T cells. *Biomolecules*.

[28] Wherry E. J., Kurachi M. (2015). Molecular and cellular insights into T cell exhaustion. *Nature Reviews Immunology*.

[29] Wu X., Zhang H., Xing Q. (2014). PD-1^+^ CD8^+^ T cells are exhausted in tumours and functional in draining lymph nodes of colorectal cancer patients. *British Journal of Cancer*.

[30] McGeachy M. J., Cua D. J., Gaffen S. L. (2019). The IL-17 family of cytokines in health and disease. *Immunity*.

[31] Luo W., Tian L., Tan B. (2022). Update: innate lymphoid cells in inflammatory bowel disease. *Digestive Diseases and Sciences*.

[32] Wu H. X., Jin L. P., Xu B., Liang S. S., Li D. J. (2014). Decidual stromal cells recruit Th17 cells into decidua to promote proliferation and invasion of human trophoblast cells by secreting IL-17. *Cellular & Molecular Immunology*.

[33] Kumar R., Theiss A. L., Venuprasad K. (2021). ROR*γ*t protein modifications and IL-17-mediated inflammation. *Trends in Immunology*.

[34] Ivanov I. I., McKenzie B. S., Zhou L. (2006). The orphan nuclear receptor RORgammat directs the differentiation program of proinflammatory IL-17^+^ T helper cells. *Cell*.

[35] Cua D. J., Tato C. M. (2010). Innate IL-17-producing cells: the sentinels of the immune system. *Nature Reviews. Immunology*.

[36] Wei L., Laurence A., Elias K. M., O’Shea J. J. (2007). IL-21 is produced by Th17 cells and drives IL-17 production in a STAT3-dependent manner. *Journal of Biological Chemistry*.

